# DIFFUSE PLANE XANTHOMATOSIS IN A PATIENT WITH BUDD-CHIARI SYNDROME AND MONOCLONAL GAMMOPATHY

**DOI:** 10.4103/0019-5154.57616

**Published:** 2009

**Authors:** Mukadder Koçak, Hatice Keleş, Fahri Yakaryilmaz, Önder Bozdoğan, Sefa Güliter

**Affiliations:** *From the Department of Dermatology, University of Kirikkale, School of Medicine, Ankara, Turkey.*; 1 *From the Department of Internal Medicine, University of Kirikkale, School of Medicine, Ankara, Turkey.*; 2 *From the Department of Gastroenterology, University of Kirikkale, School of Medicine, Ankara, Turkey.*; 3 *From the Department of Pathology, University of Kirikkale, School of Medicine, Ankara, Turkey.*

**Keywords:** *Budd-Chiari syndrome*, *diffuse plane xanthoma*, *monoclonal gammopathy*

## Abstract

Diffuse plane xanthomas are characterized by the presence of yellowish plaques on the eyelids, neck, upper trunk, buttocks, and flexural folds. Histology shows foamy histiocytes in the dermis. Approximately half of the cases are associated with lymphoproliferative disorders. Budd-Chiari syndrome is an uncommon condition induced by thrombotic or nonthrombotic obstruction of hepatic venous outflow. We present a case of diffuse plane xanthoma in a 62-year-old man who developed normolipemic plane xanthomas coinciding with Budd-Chiari syndrome and monoclonal gammopathy. We review the English-language literature regarding the rare association of xanthomas and Budd-Chiari syndrome.

## Introduction

Budd-Chiari syndrome is rare, but its exact frequency is unknown. The syndrome most often occurs in patients with underlying thrombotic diathesis, including myeloproliferative disorders, such as polycythemia vera and paroxysmal nocturnal hemoglobinuria, pregnancy, tumors, chronic inflammatory diseases, clotting disorders, and infections. Diffuse plane xanthomatosis (DPX) was first described by Altman and Winkelmann in 1962.[1] In 1966, Lynch and Winkelmann recognized the relationship of DPX to diseases of the reticuloendothelial system.[2] Since then, several cases of DPX associated particularly with multiple myeloma and monoclonal gammopathy have been reported.[1–8]

## Case Report

A 60-year-old male patient was admitted to the gastroenterology clinic in June 2004 with a three-year history of complaints of fatigue, itching and palpitation, which had been diagnosed as monoclonal gammopathy. The patient was referred to our clinic for consultation about the itching. It was determined that the present rash had appeared[9,10] years ago and that it was localized only to the face, periorbital region, and forehead in initial years, increasing continuously in the last two years. His family history was negative for hyperlipidemia and xanthoma.

**Figure 1:**
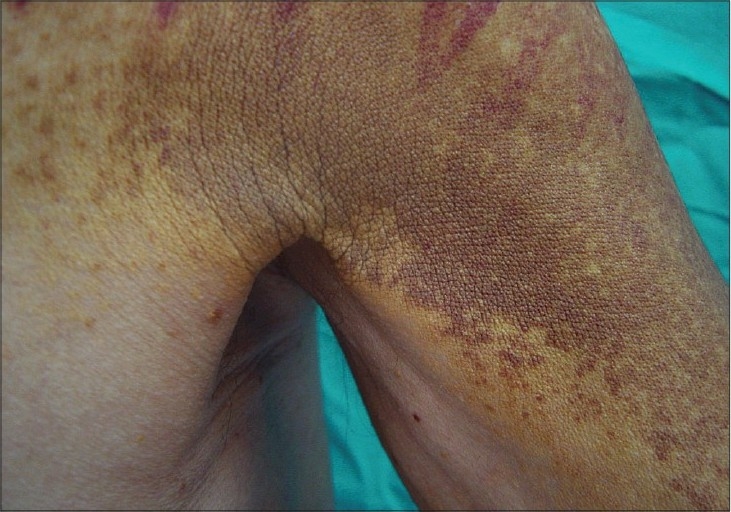
Confluent yellow-brown plaques in the lower extremity with a linear configuration

**Figure 2:**
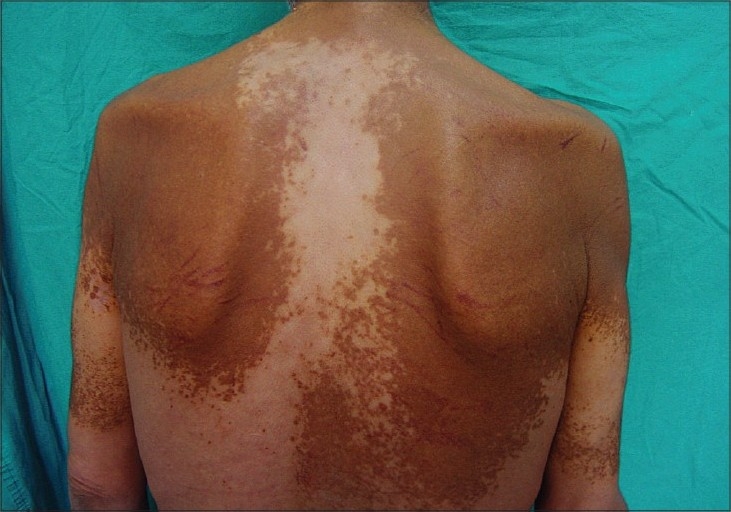
Large xanthomatous plaque on the back

**Figure 3:**
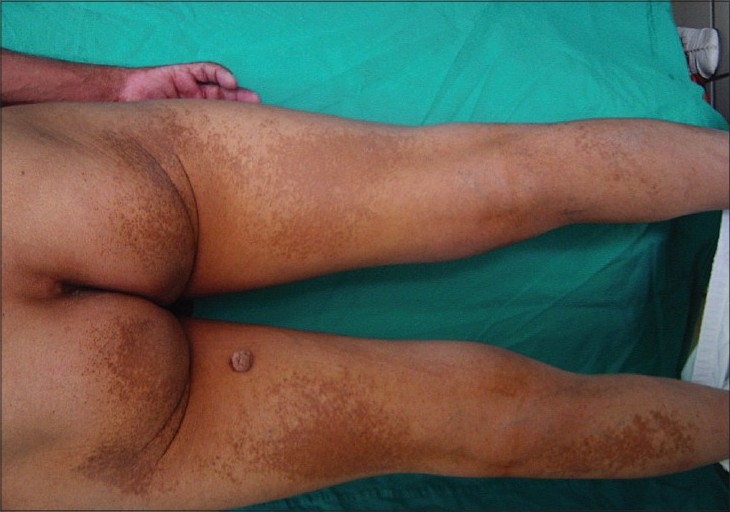
Bruise over the plaques owing to severe and persistent itching

**Figure 4:**
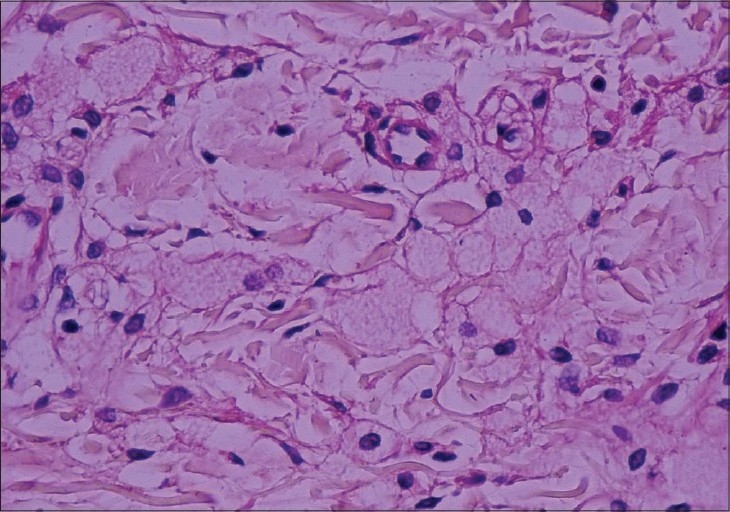
Biopsy specimen. Foam histiocytes infiltrating the dermis and perivascular area (H and E, ×200)

Physical examination revealed flat, slightly infiltrated yellow-orange and yellow-brown plaques covering the forehead, eyelids, preauricular area, neck, proximal arms, upper trunk, buttocks, and lower extremity. Identical plaques were located in the lower and upper extremity in a more linear configuration [Figures 1 and 2] There were bruises over the plaques owing to severe and persistent itching [Figure 3]. Histological examination of skin biopsy revealed an infiltrate of foamy macrophages in the papillary dermis and perivascular region [Figure 4]. The foamy cells were negative for S-100 and CD1a antibodies. The overlying epidermis was normal. IgA, IgG, IgM, C3, and C4 were established to be negative in direct immunofluorescence investigations.

Laboratory investigations showed: Hemoglobin, 11.2g/ dl; WBC, 8900/mm^3^; ESR, 64 mm/h; total protein, 7.6 (4.5-7.6); serum albumin, 2.4 (2.5-4.5); platelet count, 75000/mm^3^; liver enzymes SGOT, 74 (0-40); SGPT, 56 (0-45); GGT, 64 (0-60); Apo A, 180 mg/dl (73-169); and Apo B, 143 mg/dl (58-138). Values for renal and thyroid function tests and autoantibodies, serum electrolytes, cryoglobulins, and alkaline phosphatase were within normal ranges or negative. There was no Bence Jones proteinuria. A bone marrow biopsy specimen and aspirate showed marked plasma cell proliferation. Liver biopsy specimen showed no abnormality. Serum cholesterol and triglycerides were assayed in fasting serum and showed: Triglycerides, 277 mg/dL (50-150); cholesterol 344 mg/dL (0-200); high- density lipoprotein (HDL), 58 mg/dL (45-65); and low-density lipoprotein (LDL), 228 mg/dL (0-130). Serum levels of IgA, IgM, IgE and IgG were normal. λ light chain and λ light chain in the urine were detected as 57.5 (0-18.5) and 50.0 (0-50), respectively, while these values were 2320 (629-1350) and 723 (313-723), respectively, in the serum. Serum immunofixation electrophoresis was evaluated as IgG λ light chain while in urine immunofixation electrophoresis detected λ light chain. Serum immuno- electrophoresis revealed a monoclonal IgG Κ protein, a finding interpreted as a monoclonal gammopathy of undetermined significance.

The patient was admitted again in 2005 due to upper gastrointestinal tract (GIT) bleeding. Endoscopy of upper GIT was carried out, which detected varices in the esophagus and fundus. Abdominal magnetic resonance imaging (MRI) angiogram detected a thrombus at the level of diaphragm in inferior vena cava inferior and thrombi were observed in hepatic veins in hepatic venography. The patient was diagnosed with Budd-Chiari syndrome and progressive increase was established in skin lesions.

## Discussion

Patients with DPX exhibit large flat, plaque-like xanthomatous skin lesions involving the eyelids, neck, upper trunk, buttocks, and flexures. Plane xanthomas have been separated into two groups. Group I is associated with increased serum levels of lipids because of familial hyperlipidemia. Group II has either normal or slightly increased lipid levels without any family history. Group II can be subdivided into three groups, as idiopathic, underlying disease-associated and abnormalities of the structure or content of lipoproteins.[6,9,10] Lipid metabolism is usually normal, and an association with underlying diseases has been considered to be high.[1–3] It is most frequently associated with the diseases multiple myeloma and monoclonal gammopathy. However, DPX has been associated with chronic granulocytic or lymphatic leukemia, Waldenström's macroglobulinemia, cryoglobulinemia, lymphoma, Sézary syndrome, Castleman's disease, histiocytosis X, exfoliative dermatitis, actinic reticuloid, and photosensitive eczema.[1,3,5–7,10] Protein abnormalities, urticaria, angioedema, and complement deficiency may be seen in such patients.[10] Marcoval *et al*. carried out a clinico- pathological study to determine the incidence of associated disorders in eight patients with DPX. It was found that three of the eight patients had a reticuloendothelial disease (benign monoclonal gammopathy in 2 and chronic myelomonocytic leukemia in 1). They suggested that the incidence of underlying disease associated with DPX seems to be lower than expected.[3]

The pathogenesis in DPX has not been clearly defined.[1,2,5] In cases associated with gammopathy, it is postulated that paraprotein-lipoprotein complexes (IgG-LDL) may be recognized as modified LDL by scavenger receptors on macrophages, resulting in development of cutaneous xanthomas. Other authors consider that DPX is a histiocytosis-derived xanthomatosis in the spectrum of non-X histiocytosis. Possible explanations include the secretion of cytokines or immunoglobulins by the underlying lymphocytic proliferation, which in turn could stimulate macrophages or alter lipoprotein activity.[5,8] Our patient reported having periorbital xanthelasma for about 10 years. These lesions had been silent until the monoclonal gammopathy was detected, after which they spread to other sites on the face, neck, and upper chest immediately after the monoclonal gammopathy was activated.

Histological examination reveals large sheets and clusters of foamy cells, single and in small groups, diffusely scattered throughout the dermis, although occasionally they may appear predominantly in a perivascular location. Although some authors consider that Touton giant cells are rarely present in DPX, they have been observed in several cases. Histopathologic features observed in our cases are consistent with those in DPX. Histopathologic pattern was not related to the presence or absence of an underlying disease. The immune complex is deposited in the skin. There are opinions supporting or challenging this.[3,5–7] In our case, we could not determine immune complex deposition.

The monoclonal gammopathy is characterized by clonal proliferation of plasma cells that produce a homogeneous immunoglobulin protein. The diagnosis of many plasma cell dyscrasias is facilitated by detection of the M protein in serum or urine. M protein is an IgG type in most cases, with predominance of IgG Κ and IgG λ.[7,8, ^12^] In our case, lipid levels were also slightly high and monoclonal gammopathy was concurrent.

Budd-Chiari syndrome is an uncommon condition induced by thrombotic or nonthrombotic obstruction of hepatic venous outflow. Hepatomegaly, ascites, and abdominal pain are its most characteristic findings. Obstruction of intrahepatic veins leads to congestive hepatopathy. Most patients have an underlying thrombotic diathesis. Causes of Budd-Chiari syndrome are as follows: Polycythemia rubra vera, paroxysmal nocturnal hemoglobinuria, unspecified myeloproliferative disorder, antiphospholipid antibody syndrome, essential thrombocytosis, etc.

Patients with DPX must be clinically followed because it can precede the occurrence of an associated condition.[5] In our case, monoclonal gammopathy and Budd-Chiari syndrome coexisted. In literature reviews, no case of xanthoma accompanying Budd-Chiari was seen. In our case, xanthoma initially developed and monoclonal gammopathy diagnosis was made in 2004, and rapid increase in lesions was determined. In 2006, the patient was diagnosed with Budd-Chiari syndrome. The presence of myeloproliferative diseases in the pathogenesis of both Budd-Chiari syndrome and DPX indicates that the coexistence of these three diseases is not a coincidence. We think that this accounts for the combination of monoclonal gammopathy with both Budd-Chiari and DPX.
